# Cross-validation of an algorithm detecting acute gastroenteritis episodes from prescribed drug dispensing data in France: comparison with clinical data reported in a primary care surveillance system, winter seasons 2014/15 to 2016/17

**DOI:** 10.1186/s12874-019-0745-5

**Published:** 2019-05-31

**Authors:** Ana-Maria Vilcu, Thierry Blanchon, Laure Sabatte, Cécile Souty, Milka Maravic, Thomas Hanslik, Olivier Steichen

**Affiliations:** 1 Sorbonne Université, INSERM, Institut Pierre Louis d’épidémiologie et de Santé Publique (IPLESP UMRS 1136), F-75012 Paris, France; 2 Real World Insight, IQVIA, F-92099 La Défense Cedex, France; 3 Assistance Publique - Hôpitaux de Paris (APHP), hôpital Lariboisière, Service de Rhumatologie, F-75010 Paris, France; 4 Université de Versailles Saint-Quentin-en-Yvelines, UVSQ, UFR de Médecine, F-78000 Versailles, France; 5 Assistance Publique - Hôpitaux de Paris (APHP), hôpital Ambroise Paré, Service de Médecine Interne, F-92100 Boulogne Billancourt, France; 6 Sorbonne Université, Inserm, université Paris 13, Laboratoire d’informatique médicale et d’ingénierie des connaissances en e-santé, LIMICS, F-75006 Paris, France; 7 Assistance Publique - Hôpitaux de Paris (APHP), hôpital Tenon, Service de Médecine Interne, F-75020 Paris, France

**Keywords:** Gastroenteritis, Epidemiology, Public health surveillance, Sentinel surveillance, Pharmacy, Primary care physicians

## Abstract

**Background:**

This study compares an algorithm to detect acute gastroenteritis (AG) episodes from drug dispensing data to the validated data reported in a primary care surveillance system in France.

**Methods:**

We used drug dispensing data collected in a drugstore database and data collected by primary care physicians involved in a French surveillance network, from season 2014/15 to 2016/17. We used an adapted version of an AG discrimination algorithm to identify AG episodes from the drugstore database. We used Pearson’s correlation coefficient to evaluate the agreement between weekly AG signals obtained from the two data sources during winter months, in the overall population, by specific age-groups and by regions.

**Results:**

Correlations between AG signals for all ages were 0.84 [95%CI 0.69; 0.92] for season 2014/15, 0.87 [95%CI 0.75; 0.93] for season 2015/16 and 0.94 [95%CI 0.88; 0.97] for season 2016/17. The association between AG signals estimated from two data sources varied significantly across age groups in season 2016/17 (*p*-value < 0.01), and across regions in all three seasons studied (*p*-value < 0.01).

**Conclusions:**

There is a strong agreement between the dynamic of AG activity estimated from drug dispensing data and from validated primary care surveillance data collected during winter months in the overall population but the agreement is poorer in several age groups and in several regions. Once automated, the reuse of drug dispensing data, already collected for reimbursement purposes, could be a cost-efficient method to monitor AG activity at the national level.

**Electronic supplementary material:**

The online version of this article (10.1186/s12874-019-0745-5) contains supplementary material, which is available to authorized users.

## Background

Acute gastroenteritis (AG) is a common infectious intestinal disease, causing acute diarrhoea or vomiting [1]. Viruses are the most common causal agents, followed by bacteria and parasites [2–4]. In temperate climates, peaks of AG activity occur every winter [4–6], causing substantial morbidity and economic burden in high resource countries and high mortality in low resource countries, particularly in children [7–9] .

In France, based on cases reported by general practitioners (GPs) registered to the Sentinelles network, weekly AG incidences are estimated at a regional and national level, in the whole population and by age groups, since 1990. These estimates are shared in real-time with public health authorities in order to detect outbreaks and implement timely control measures [10].

A study investigating healthcare-seeking behaviors for AG in the French population has shown that 33.4% of individuals having an AG episode would consult a physician (of which 92.8% would consult a GP), and 96% would buy prescribed medications [9, 11].

Drug dispensing data have become a valuable tool for detecting and monitoring disease occurrence in the last decades, especially when availability of clinical data is limited [12]. Diagnoses can be inferred from prescribed pharmaceutical dispenses when drugs or associations of drugs are disease-specific. However, the use of drug dispensing data can be challenging when studying diseases with less specific drug treatment such as AG, for which drugs and dosages depend on patients’ symptoms and characteristics (age, weight).

Bounoure et al. have designed an algorithm meant to identify AG episodes from drug dispensing data included in the SNIIRAM, the French administrative health care claims database [13, 14]. Based on the delay between drug prescription and drug dispense, the age of patients, the type, volume and number of classes of drugs dispensed, this algorithm identifies dispenses intended for AG treatment among all dispenses containing drugs potentially used in AG treatment. This algorithm has been designed and implemented only in the SNIIRAM database and has been validated through surveys in drugstores by auditing prescriptions at the origin of drug dispenses included in the analysis [13]. An early version of the algorithm has been cross-validated by comparing its results to data from the Sentinelles Network but this work has not been published [15].

The aim of our study is to compare the AG activity dynamic estimated with an updated version of the discrimination algorithm [16] applied on an independent drug dispensing database to the AG activity dynamic estimated from data reported by the Sentinelles GPs (SGPs).

## Methods

### Data sources

#### The LTD database

The LTD database contains all anonymised medication dispenses prescribed in ambulatory care and purchased in a panel of approximatively 7000 French drugstores, since 2012. The drugstores included in the panel represent nearly 30% of all drugstores and are representative in terms of geographical spread in continental France and age of population covered [17, 18].

Data collected includes: dispensed drugs characteristics - such as packaging, dosage, International Nonproprietary Name (INN), EphMRA code (European Pharmaceutical Market Research Association), CIP code (Code Identifiant de Présentation – a unique code taking into account drug packaging), dispensed volume, prescription and dispensing dates, county of the dispensing drugstore; patient characteristics - a unique anonymised identifier (id), year of birth and gender; prescriber characteristics - such as speciality and geographical area.

#### The French Sentinelles network

The French Sentinelles network is a surveillance system established in 1984 and based on the voluntary participation of 1395 primary care physicians in continental France (representing ~ 2% of all French GPs) [10]. Sentinel physicians collect several indicators among which acute diarrhoea (since 1990). The monitoring of AG activity is based on SGPs acute diarrhoea case report using the following case definition: *recent acute diarrhoea (at least 3 daily watery or nearly so stools, for less than 14 days) motivating consultation* [19]. Weekly AG incidences are then estimated at a regional level and pooled to provide a national incidence estimate [20].

### The AG discrimination algorithm

#### Description

The algorithm consists in several steps based on the definition of three drug categories [13, 15]: drugs usually prescribed for AG treatment (further called “AG usual drugs”); other drugs frequently prescribed for AG treatment (further called “AG possible drugs”); when AG usual or possible drugs are prescribed to treat another condition, they may be prescribed with other drugs that are never used to treat AG (further called “AG excluding drugs”).

AG usual drugs include oral rehydration salts (ORS), which are highly specific of AG but only prescribed in children, and anti-emetics, probiotic antidiarrhoeals, intestinal antipropulsives, intestinal absorbents and intestinal anti-infectious agents, which are very often prescribed for AG but sometimes for other conditions. AG possible drugs include antispasmodic agents. The detailed list of AG usual drugs and AG possible drugs is presented in Additional file 1.

AG excluding drugs are: antibiotics, antineoplastic agents used in cancer therapy, gastric antacids, drugs used for inflammatory bowel diseases and anti-emetics in injectable form (Additional file 2). Contrary to the original algorithm, we did not include drugs for peptic ulcer and gastro-oesophageal reflux disease (ATC A02B) among excluding drugs in this study, because proton pomp inhibitors (PPI) are prescribed to many patients in France (about 20% of patients over 45 years old have been prescribed PPI at least once in 2016 in France [21]).

#### Implementation

The AG discrimination algorithm was applied to drug dispensing data recorded in the LTD database. Pharmaceutical specialties belonging to one of the three above-mentioned drug categories were identified and extracted from the LTD database based on either the CIP code, the INN or their commercial name.

AG is a self-limited disease and drugs are thus typically prescribed for 3 to 7 days. Therefore, for each AG usual or possible drug, a GP defined the maximum volume (number of boxes) consistent with AG treatment. Larger amounts were considered as not indented for AG treatment (treatment for another disease, home drug stock or travel packing medications).

Then, all drug dispenses containing at least one AG usual drug were extracted from the LTD database. The extraction was limited to drug dispenses associated to prescriptions issued by a GP for which prescription date and patient’s age were available.

According to the AG discrimination algorithm, drug dispenses were classified as intended to treat an AG episode if the time lag between consultation and drug dispense was < 2 days (patients who consult their GP for AG buy the prescribed medicine without delay in order to alleviate its disabling symptoms) and one of the four conditions below were fulfilled:

A. dispensing of an ORS; or
B. dispensing of at least three categories of AG possible drugs; or
C. dispensing of a consistent volume of at least two categories of AG possible drugs, without excluding drug; or
D. age ≤ 15 years and dispensing of a consistent volume of an intestinal anti-propulsive or an anti-emetic, without excluding drug and no more than 4 drugs dispensed.

#### Cross-validation: comparison with Sentinelles data

We compared AG activity estimated through the discrimination algorithm applied on drug dispensing data to AG activity estimated from primary care Sentinelles data. Since AG surveillance is intended to detect the winter outbreak, main analyses were limited to the “winter” period to which we will further refer as “season”. Analyses on the “summer” period are reported as secondary analyses.

The winter period was considered to last from week number 36 of year N to week number 15 of year N + 1, as defined by the International Organization for Standardization (ISO) [22], while the summer period was considered to last from week number 16 of year N + 1 to week number 35 of year N + 1.

The indicator for AG activity estimated from drug dispensing data was the weekly number of AG episodes identified by the discrimination algorithm in the whole population attending LTD drugstores. Analyses were carried out separately for winter seasons 2014/15, 2015/16 and 2016/17. Multiple identified AG episodes per patient were considered independent if the delay between two consecutive AG dispenses exceeded 15 days; otherwise, the first dispense was counted as an AG episode and the subsequent one was considered a treatment renewal. In order to reduce the risk of misclassifying other chronic treatments including AG drugs as AG episodes, patients for whom the algorithm detected more than 3 AG episodes per season were excluded.

The indicator for AG activity estimated from Sentinelles data was the weekly national AG incidence estimated by the Sentinelles Network from the clinical AG cases reported by the SGPs.

Both indicators are not expressed on the same scale: number of cases identified among an unknown number of individuals who buy or would buy their prescription drugs in a LTD drugstore for the indicator from drug dispensing data; estimated national incidence for the indicator from Sentinelles data. As a consequence, usual statistical methods of agreement assessment (intraclass correlation coefficient, Bland and Altman plots) could not be used. Correlations between the weekly AG incidence estimated by the Sentinelles Network and the weekly number of AG episodes identified by the algorithm using drug dispensing data were evaluated within each week using Pearson’s correlation coefficient. In order to formally test the linearity assumption, the weekly number of AG episodes identified by the algorithm using drug dispensing data was regressed against the weekly AG incidence estimated by the Sentinelles Network, using a linear model and a cubic spline regression model with 3 knots, which were compared through analysis of variance (ANOVA).

There is some uncertainty regarding the date of GP consultation related to an AG drug dispense or to a Sentinelles AG report. Drugs prescribed for AG by the GP may indeed be dispensed a few days later. To account for the potential postponement of drug dispenses, correlations were also estimated at one week lag between Sentinelles data (week n) and drugs dispensing data (week n + 1). On the other hand, the exact consultation date of Sentinelles AG cases is unknown; each SGP reports the number of AG cases seen in a time window of maximum 12 days which are then evenly distributed over the entire time window covered and a number of weekly cases is imputed. In order to account for the potential postponement of Sentinelles data, we also computed correlations at one week lead between Sentinelles data (week n + 1) and drug dispensing data (week n).

Subgroup analyses were then performed by age groups (0–4 years, 5–14 years, 15–64 years and 65 years or older) on one hand, and by regions on the other hand. Age-related subgroup analysis could not be performed for season 2014/15 because prescriptions issued for children aged less than 2 years were often recorded on the parents’ account instead of the child’s account in the LTD database up to 2014. Geographical subgroup analysis was performed at the regional level (NUTS2 level - Nomenclature of Statistical Territorial Units Level 2; 13 regions in continental France) [23]. Analysis of variance (ANOVA) with interaction terms was used to test for differences in the association of the two outcomes across subgroups.

In order to assess the size of the weekly AG incidence detected by the algorithm within the LTD sample of drugstores with respect to the AG Sentinelles incidence estimated at the national level, we computed weekly rates between the number of new AG episodes identified from drug dispensing data and the national number of AG cases estimated from Sentinelles data. Additionally, we compared the dates of peak of AG activity observed from the two data sources.

All analyses were performed using R version 3.3.3 [24].

## Results

AG surveillance season 2014/15 lasted from September 1st 2014 to April 12th 2015, season 2015/16 lasted from August 31st 2015 to April 17th 2016 and season 2016/17 lasted from September 5th 2016 to April 16th 2017.

**Fig. 1:**
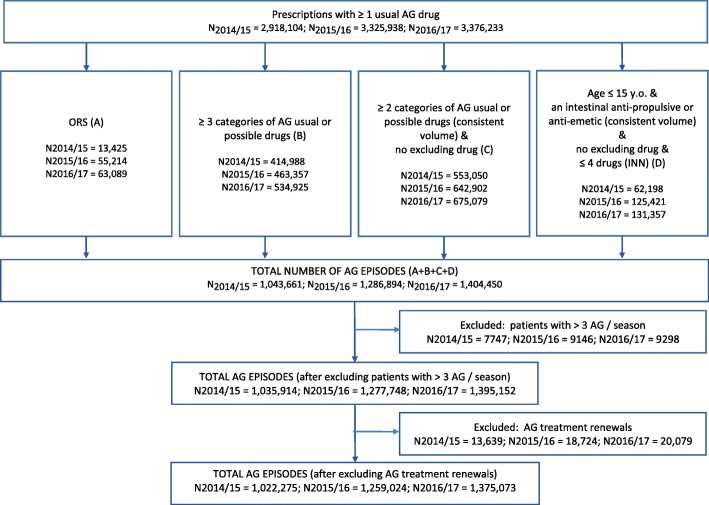
Identification of acute gastroenteritis episodes among prescriptions containing drugs used in the treatment of acute gastroenteritis, according to the discrimination algorithm applied on drug dispensing data available in the LTD database, seasons 2014/15 to 2016/17 (week number 36 of year N to week number 15 of year N + 1)

Overall, 2,918,104 drug dispenses potentially intended for AG treatment were identified in 2014/15, 3,325,938 in 2015/16 and 3,376,233 in 2016/17. Among them, 1,022,275 (35%) were classified as intended to treat an AG episode in 2014/15, 1,259,024 (38%) in 2015/16 and 1,375,073 (41%) in 2016/17 (Fig. 1). The numbers of AG episodes detected by age group are presented in Table 1.

**Table 1:** Number of AG cases estimated from drug dispensing data and from the Sentinelles network, winter seasons 2014/15 to 2016/17 (week number 36 of year N to week number 15 of year N + 1)

Season	Age group (years)	Cases detected from drug dispensing data N (%)	Cases estimated from Sentinelles data N (%)
2014/15^a^	All ages	1,022,275 (100)	3,333,785 (100)
2015/16	All ages	1,259,024 (100)	3,050,341 (100)
	0–4	202,605 (16)	486,901 (16)
	5–14	267,481 (21)	526,054 (17)
	15–64	656,346 (52)	1,827,082 (60)
	65+	132,592 (11)	210,304 (7)
2016/17	All ages	1,375,073 (100)	3,281,268 (100)
	0–4	232,200 (17)	534,283 (16)
	5–14	290,084 (21)	550,677 (17)
	15–64	720,518 (52)	1,984,851 (60)
	65+	132,271 (10)	211,457 (6)

^a^ Due to limitations of the drug dispensing database, the analysis at age group level could not be carried out for season 2014/15

Nearly 93% of individuals identified from drug dispensing data each season had a single AG episode, while 6% had two AG episodes (Additional file 3).

**Fig. 2:**
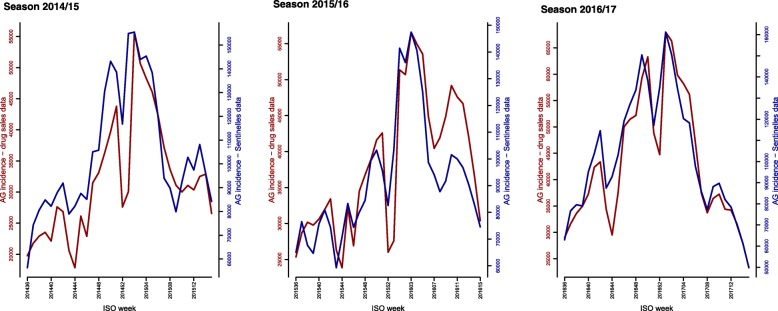
Weekly number of acute gastroenteritis episodes estimated from drug dispensing data using the discrimination algorithm vs. weekly incidences of acute gastroenteritis estimated at the French Sentinelles Network, in the overall population, seasons 2014/15 to 2016/17 (week number 36 of year N to week number 15 of year N + 1)

**Fig. 3:**
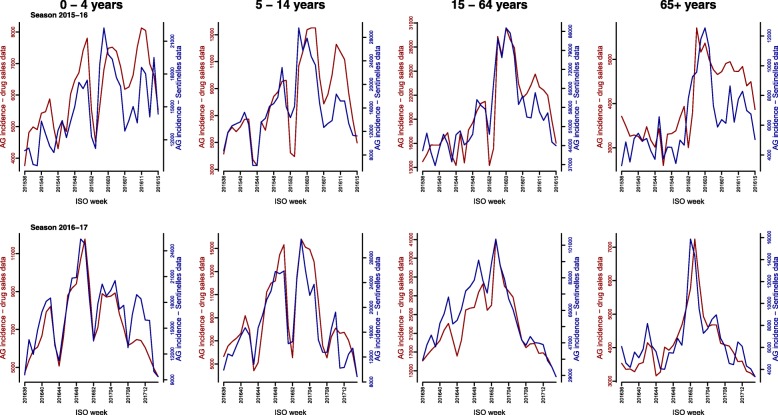
Weekly number of acute gastroenteritis episodes estimated from drug dispensing data using the discrimination algorithm vs. weekly incidences of acute gastroenteritis estimated at the French Sentinelles Network, per age groups, seasons 2015/16 and 2016/17 (week number 36 of year N to week number 15 of year N + 1)

The best correlations between AG activity estimated from drug dispensing data and AG activity estimated from Sentinelles data occurred at one week lag for season 2014/15, and at no lag for seasons 2015/16 and 2016/17 (Figs. 2 and 3). However, results at no lag and at one week lag were very close for season 2014–2015 and, for the sake of consistency, all results are reported at no lag.

Overall correlations between both estimates of AG activity were 0.84 [95%CI 0.69; 0.92] for season 2014/15, 0.87 [95%CI 0.75; 0.93] for season 2015/16 and 0.94 [95%CI 0.88; 0.97] for season 2016/17. No statistically significant differences were observed between the linear models and the non-parametric models regressing the AG activity estimated from drug dispensing data against AG activity estimated from Sentinelles data (Additional file 4: Figure S1).

The association between AG activity estimated from drug dispensing data and AG activity estimated from Sentinelles data differed significantly across age groups in season 2016/17 (*p*-value < 0.01), but not in season 2015/2016 (p-value = 0.14). In season 2016/17, the highest correlation was observed in the group of 15–64 year olds (0.92 [95%CI 0.84; 0.96]) while the lowest correlation was observed in the group of elderly (0.87 [95%CI 0.74; 0.93]). Full details are available in Table 2 and Additional file 5.

**Table 2:** Pearson’s correlations and weekly rates of the number of acute gastroenteritis episodes estimated from drug dispensing data with respect to the number of acute gastroenteritis episodes estimated from Sentinelles data, winter seasons 2014/15 to 2016/17 (week number 36 of year N to week number 15 of year N + 1)

Season	Age groups (years)	Correlation coefficient^a^ [95% CI]	Median weekly rates (%) [1st, 3rd quartiles]
2014/15^b^	All ages	0.84 [0.69; 0.92]	44 [38; 47]
2015/16	All ages	0.87 [0.75; 0.93]	44 [43; 49]
	0–4	0.75 [0.54; 0.87]	43 [39; 45]
	5–14	0.80 [0.63; 0.90]	49 [45; 52]
	15–64	0.87 [0.75; 0.93]	37 [35; 39]
	65+	0.75 [0.55; 0.87]	68 [60; 76]
2016/17	All ages	0.94 [0.88; 0.97]	45 [39; 48]
	0–4	0.91 [0.82; 0.95]	44 [40; 47]
	5–14	0.90 [0.81; 0.95]	52 [44; 57]
	15–64	0.92 [0.84; 0.96]	37 [34; 40]
	65+	0.87 [0.75; 0.93]	64 [58; 76]

^a^ Correlation are computed at no time lag between the weekly acute gastroenteritis incidences estimated using the discrimination algorithm applied on drug dispensing data available in the LTD database and the weekly acute gastroenteritis incidences estimated at the Sentinelles Network

^b^ Due to limitations of the drug dispensing database, the analysis at age group level could not be carried out for season 2014/15

The association between AG activity estimated from drug dispensing data and AG activity estimated from Sentinelles data differed significantly across regions in the three studied seasons (p-value < 0.01). Correlations ranged from 0.45 [95%CI 0.11; 0.69] to 0.83 [95%CI 0.67; 0.91] in season 2014/15, from 0.43 [95%CI 0.10; 0.67] to 0.83 [95%CI 0.67; 0.91] in season 2015/16, and from 0.49 [95%CI 0.17; 0.72] to 0.89 [95%CI 0.78; 0.94] in season 2016/17, all age groups pooled (Additional file 6).

The median rates of the weekly number of AG episodes estimated from drug dispensing data with respect to the national number of AG episodes estimated from Sentinelles data are reported in Table 2. For each season, both indicators detected the same week of peak of AG activity (ISO weeks 2015w02, 2016w03 and 2017w01).

During summer periods, overall correlations between the two estimates of AG activity were 0.06 [95%CI -0.39; 0.49] in summer 2014/15, 0.67 [95%CI 0.33; 0.86] in summer 2015/16 and 0.34 [95%CI -0.12; 0.68] in summer 2016/17.

## Discussion

### Summary of results

The AG epidemic dynamic estimated from drug dispensing data through a discrimination algorithm is very similar to the AG epidemic dynamic estimated from Sentinelles clinical data, in the overall population [16].

Pearson’s correlation coefficients ranged from 0.83 to 0.94 in the whole population, but varied significantly across age groups in season 2016/17 and across regions in all three seasons studied. The seasonal median rates of the weekly number of AG cases detected by the algorithm with respect to the national incidence estimated from Sentinelles data were 45%, but higher rates were observed in patients over 65 years old, suggesting an overestimation of cases in this age group. Correlation between the two methods was weak during summer months.

### Interpretation and comparison with previous studies

An early version of the algorithm has been cross-validated by comparing AG signals estimated from the SNIIRAM database to those estimated from validated sources. AG signal estimated by the algorithm from the SNIIRAM database was close to those estimated by the Sentinelles Network (0.83 < r < 0.95, seasons 2007/08–2010/11) and by field investigations of six epidemics [15]. AG signal obtained by the algorithm from the SNIIRAM database has also been compared to data obtained from two cohort studies conducted during two waterborne disease outbreaks in 2010 and 2012. Results were close with one of the study but much less with the other, highlighting the need of more comparative studies [11].

The slightly lower correlation we observed in season 2014/15 (0.84) could be due to changes in prescription habits, such as recommendations issued in 2014 to restrict domperidone use in nausea and vomiting treatment [25]. Another explanation could be the limitation of the LTD database in correctly recording drug dispenses intended for children aged one year or less.

Strong correlations were observed in the age group-specific analyses as well. Significant differences in the strength of association were observed across age groups in season 2016/17, but not in season 2015/16. The slightly lower correlation observed in the group of elderly could be due to an overestimation of the number of AG episodes identified from drug dispensing data. Individuals aged 65 years and over indeed often suffer from several comorbidities and are prescribed more drugs on the long run. Thus, the algorithm may misclassify as AG episodes drug dispenses containing a large number of drugs, including AG possible drugs. A higher proportion of patients with more than 3 AG episodes per season was observed in this age group (*p*-value < 0.01 compared to other age groups), supporting the hypothesis of an overestimation of AG cases due to drugs prescribed on the long run. Additionally, median rates of weekly AG incidence estimated from drug dispensing data expressed as a percentage of national AG Sentinelles incidences were highest in the group of elderly (64% in season 2015/16 and 68% in season 2016/17). Since the panel of LTD drugstores represents approximately one third of the total of drugstores in continental France, on average, we would expect a weekly rate of ~ 30%, which is the case in the age group 15 to 64 years old, for which the detection algorithm is probably the most accurate.

Correlations vary significantly across regions and have wider confidence intervals. However, the method to which we compare outcomes produced by the algorithm is not perfect. The precision of Sentinelles incidences at the regional level depends on SGPs participation, which differs across regions. In order to minimize biases that may arise due to the fact that the monitored population is not spatially representative of the general French population, the AG Sentinelles incidence is first estimated by region and then pooled to estimate national incidence [20].

### Strengths and limitations

The main limitation of our study is the lack of a stable reference population in the LTD database, which would allow us to estimate AG incidence rates from drug dispensing data and to conduct a more robust concordance analysis. Linearity tests carried out suggested a linear relationship between the weekly AG incidence observed from Sentinelles data and the weekly number of AG cases estimated from drugs dispensing data. Scatter plots suggest a good agreement over the whole range of weekly incidence during the winter season.

We could not account for patients’ switches between LTD drugstores and non-LTD drugstores. Since no information on patients’ drug dispensing history in non-LTD drugstores is available, some treatment renewals might be misclassified as incident AG episodes. However, nearly 93% of AG patients included in the study were estimated to have a single AG episode along the respective season. Moreover, AG treatment renewals identified in the LTD database represented less than 2% of all AG episodes identified. This suggests that misclassification associated with treatment renewals would have little impact on the estimation of AG activity. In order to minimize the risk of misclassification, we have excluded patients for whom the algorithm identified more than 3 AG episodes per season as they may receive AG possible drugs as part of a chronic treatment for a disease other than AG.

Misclassification of drug dispenses might also occur due to the design of the algorithm and specific limitations of drug dispensing databases on which it is applied. In our study, analysis at the age group level was not possible before season 2015/16 due to misreporting of drug prescriptions intended for young children which could result in the misclassification of some drug dispenses among age-related subgroups and, thus, in an underestimation of the overall number of AG episodes among children and overestimation among adults. Moreover, some steps of the algorithm target only drug dispenses intended for children. Some prescriptions misreported as intended for adults would therefore be misclassified as not intended for AG. However, all ages AG activity estimated from drug dispensing data was still highly correlated to AG activity estimated from Sentinelles data, suggesting a low impact of this limitation in the overall analysis.

We have shown that AG activity estimated from drug dispenses and from Sentinelles data correlate poorly during summer. Both methods present some potential limitations in capturing AG activity during this period: potential difficulties of the algorithm in discriminating AG prescriptions from preemptive prescriptions (travel packing medications) in the drug dispensing database; a lower participation of SGPs at the AG routine surveillance, resulting in a lower precision of AG incidences in the Sentinelles database. The algorithm should therefore not be used in summer, but this is an acceptable drawback since it is intended to detect the winter outbreak of AG.

Since the algorithm was calibrated with reference to prescribing habits for AG treatment in primary care in France, its use may be considered in countries with similar AG management and prescribing habits. However, further calibration and evaluation studies are required in countries where different AG management protocols are used. Moreover, it relies on a database primarily intended for reimbursement purposes, and it could perform differently where drugs can be bought over the counter or even in places other than drugstores. For example, oral rehydration solutions can be purchased without prescription in grocery stores in several countries such as the United States.

## Conclusions

The results of our study highlight the potential of the discrimination algorithm as a tool facilitating exploitation of drug dispensing data for AG monitoring, which could help in overcoming some limitations related to the scarcity of real time AG clinical data. This version of the algorithm is currently used to identify AG cases in an ongoing study investigating the association between chronic treatment with PPI and the risk of AG during the outbreak season. Its use could be further extended to the study of additional risk factors for AG, at different geographic levels and targeting specific populations, for which clinical data would be difficult to collect.

## Additional files

Additional file 1: List of AG usual drugs and AG possible drugs used by the AG discrimination algorithm. (XLS 38 kb)

Additional file 2: List of excluding drugs used by the AG discrimination algorithm. (XLS 61 kb)

Additional file 3: Number of AG episodes per patient detected from drug dispensing data for patients included in the study, seasons 2014/15 to 2016/17 (week number 36 of year N to week number 15 of year N + 1). (PDF 39 kb)

Additional file 4: **Figure S1.** Linear model versus non-parametric model regressing the weekly number of AG cases estimated from drug dispensing data against the weekly AG Sentinelles incidence, all ages, winter seasons 2014/15 to 2016/17. (PDF 7 kb)

Additional file 5: Correlation between the weekly AG incidences estimated using the AG discrimination algorithm applied on drug dispensing data available in the LTD database and the weekly AG incidences estimated at the Sentinelles Network, overall and by age group, at no time lag, at one week of time lag and at one week of time lead between the Sentinelles data and drug dispensing data, seasons 2014/15 to 2016/17 (week number 36 of year N to week number 15 of year N + 1). (PDF 56 kb)

Additional file 6: Pearson’s correlations at region level, all age groups pooled, winter seasons 2014/15 to 2016/17 (week number 36 of year N to week number 15 of year N + 1). (PDF 45 kb)

## Abbreviations

AG: Acute Gastroenteritis
ATC: Anatomical Therapeutic Chemical Classification
CI: Confidence Interval
CIP code: Code Identifiant de Présentation
EphMRA: European Pharmaceutical Market Research Association
GP: General Practitioner
INN: International Nonproprietary Name
ISO: International Organization for Standardization
ORS: Oral Rehydration Salts
PPI: Proton Pomp Inhibitors
SGP: Sentinelles General Practitioner
