# Supplementary material for: Cross-validation of an algorithm detecting acute gastroenteritis episodes from prescribed drug dispensing data in France: comparison with clinical data reported in a primary care surveillance system, winter seasons 2014/15 to 2016/17
Source: BMC Med Res Methodol. 2019 May 31;19:110. doi: 10.1186/s12874-019-0745-5 (PMC6545010; doi:10.1186/s12874-019-0745-5)
Supplement: Supplementary file 3 — Number of AG episodes per patient detected from drug dispensing data for patients included in the study, seasons 2014/15 to 2016/17 (week number 36 of year N to week number 15 of year N + 1). (PDF 39 kb) [file 12874_2019_745_MOESM3_ESM.pdf]

Additional file 3. Number of AG episodes per patient detected from drug dispensing data for patients included in the study, seasons 2014/15 to 2016/17 (week number 36 of year N to week number 15 of year N+1)

Number of AG episodes per patient per season	Number of patients (%)		
	Season 2014/15	Season 2015/16	Season 2016/17
1	902 984 (93.4%)	1 105 267 (93.1%)	1 206 148 (93.1%)
2	57 243 (5.9%)	73 768 (6.2%)	81 023 (6.2%)
3	6 154 (0.6%)	8 315 (0.7%)	8 986 (0.7%)
