# Supplementary figures and images for: Cross-validation of an algorithm detecting acute gastroenteritis episodes from prescribed drug dispensing data in France: comparison with clinical data reported in a primary care surveillance system, winter seasons 2014/15 to 2016/17
Source: BMC Med Res Methodol. 2019 May 31;19:110. doi: 10.1186/s12874-019-0745-5 (PMC6545010; doi:10.1186/s12874-019-0745-5)

Season 2014–15

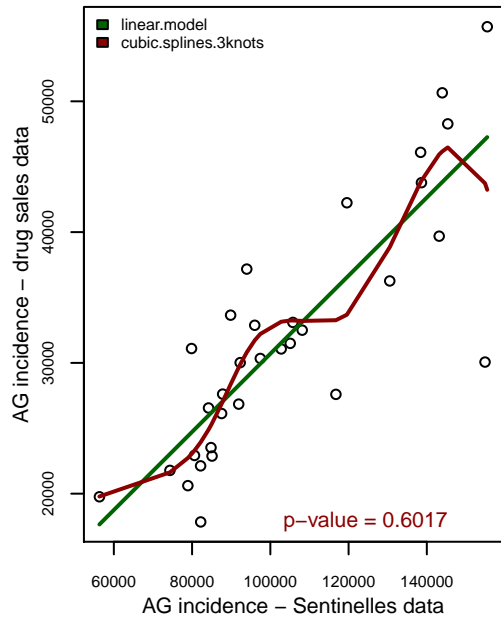

Season 2015–16

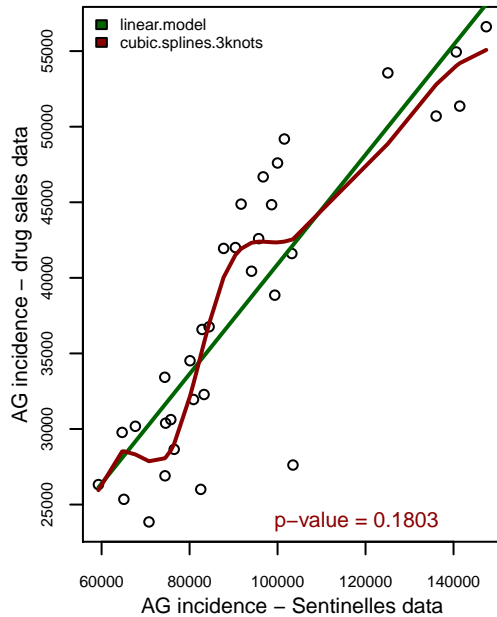

Season 2016–17

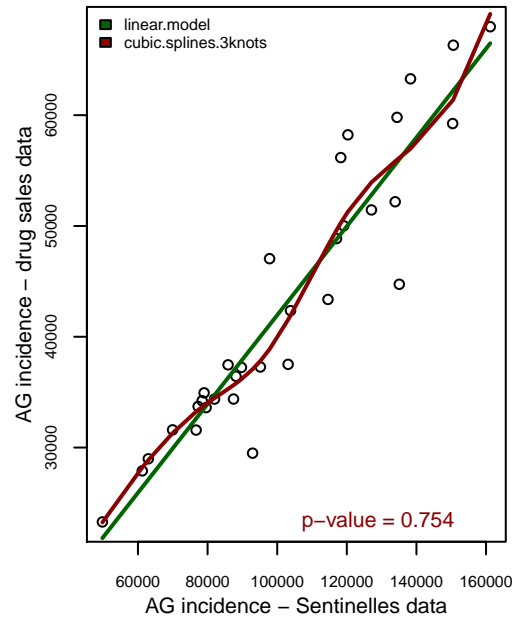

Supplement: Supplementary file 4 — Figure S1. Linear model versus non-parametric model regressing the weekly number of AG cases estimated from drug dispensing data against the weekly AG Sentinelles incidence, all ages, winter seasons 2014/15 to 2016/17. (PDF 7 kb) [file 12874_2019_745_MOESM4_ESM.pdf]
