# Supplementary material for: Cross-validation of an algorithm detecting acute gastroenteritis episodes from prescribed drug dispensing data in France: comparison with clinical data reported in a primary care surveillance system, winter seasons 2014/15 to 2016/17
Source: BMC Med Res Methodol. 2019 May 31;19:110. doi: 10.1186/s12874-019-0745-5 (PMC6545010; doi:10.1186/s12874-019-0745-5)
Supplement: Supplementary file 5 — Correlation between the weekly AG incidences estimated using the AG discrimination algorithm applied on drug dispensing data available in the LTD database and the weekly AG incidences estimated at the Sentinelles Network, overall and by age group, at no time lag, at one week of time lag and at one week of time lead between the Sentinelles data and drug dispensing data, seasons 2014/15 to 2016/17 (week number 36 of year N to week number 15 of year N + 1). (PDF 56 kb) [file 12874_2019_745_MOESM5_ESM.pdf]

Additional file 4. Correlation between the weekly AG incidences estimated using the AG discrimination algorithm applied on drug dispensing data available in the LTD database and the weekly AG incidences estimated at the *Sentinelles* Network, overall and by age group, at no time lag, at one week of time lag and at one week of time lead between the Sentinelles data and drug dispensing data, winter seasons 2014/15 to 2016/17 (week number 36 of year N to week number 15 of year N+1)

Season	Age Group	Correlations [95% CI]		
		No time lag	One week lag	One week lead
2014/15[a]	All ages	0.84 [0.69 ; 0.92]	0.87 [0.74 ; 0.94]	0.61 [0.32 ; 0.79]
2015/16	All ages	0.87 [0.75 ; 0.93]	0.84 [0.69 ; 0.92]	0.59 [0.31 ; 0.78]
	0 – 4 y.o.	0.75 [0.54 ; 0.87]	0.67 [0.42 ; 0.82]	0.47 [0.14 ; 0.70]
	5 – 14 y.o.	0.80 [0.63 ; 0.90]	0.75 [0.54 ; 0.87]	0.42 [0.09 ; 0.67]
	15 – 64 y.o.	0.87 [0.75 ; 0.93]	0.84 [0.70 ; 0.92]	0.61 [0.34 ; 0.79]
	65+ y.o.	0.75 [0.55 ; 0.87]	0.74 [0.53 ; 0.87]	0.66 [0.40 ; 0.82]
2016/17	All ages	0.94 [0.88 ; 0.97]	0.92 [0.84 ; 0.96]	0.74 [0.52 ; 0.86]
	0 – 4 y.o.	0.91 [0.82 ; 0.95]	0.75 [0.53 ; 0.87]	0.63 [0.35 ; 0.80]
	5 – 14 y.o.	0.90 [0.81 ; 0.95]	0.81 [0.64 ; 0.90]	0.54 [0.23 ; 0.75]
	15 – 64 y.o.	0.92 [0.84 ; 0.96]	0.93 [0.85 ; 0.96]	0.72 [0.50 ; 0.86]
	65+ y.o.	0.87 [0.74 ; 0.93]	0.86 [0.72 ; 0.93]	0.69 [0.45 ; 0.84]

a Due to limitations of the drug dispense database, the analysis at age group level could not be carried out for season 2014/15
