# Supplementary material for: Cross-validation of an algorithm detecting acute gastroenteritis episodes from prescribed drug dispensing data in France: comparison with clinical data reported in a primary care surveillance system, winter seasons 2014/15 to 2016/17
Source: BMC Med Res Methodol. 2019 May 31;19:110. doi: 10.1186/s12874-019-0745-5 (PMC6545010; doi:10.1186/s12874-019-0745-5)
Supplement: Supplementary file 6 — Pearson’s correlations at region level, all age groups pooled, winter seasons 2014/15 to 2016/17 (week number 36 of year N to week number 15 of year N + 1). (PDF 45 kb) [file 12874_2019_745_MOESM6_ESM.pdf]

Additional File 5. Pearson's correlations at region level, all age groups pooled, winter seasons 2014/15 to 2016/17 (week number 36 of year N to week number 15 of year N+1)

Region	Pearson's Correlation [95% CI]		
	Season 2014/15	Season 2015/16	Season 2016/17
AUVERGNE-RHONE-ALPES	0.68 [0.44 ; 0.83]	0.73 [0.52 ; 0.86]	0.88 [0.76 ; 0.94]
BOURGOGNE-FRANCHE-COMTE	0.47 [0.15 ; 0.71]	0.62 [0.35 ; 0.79]	0.81 [0.64 ; 0.90]
BRETAGNE	0.63 [0.35 ; 0.80]	0.60 [0.32 ; 0.78]	0.53 [0.22 ; 0.74]
CENTRE-VAL-DE-LOIRE	0.83 [0.67 ; 0.91]	0.70 [0.47 ; 0.84]	0.74 [0.52 ; 0.86]
CORSE	0.77 [0.57 ; 0.88]	0.57 [0.29 ; 0.77]	0.49 [0.17 ; 0.72]
GRAND EST	0.71 [0.48 ; 0.85]	0.43 [0.10 ; 0.67]	0.55 [0.25 ; 0.76]
HAUTS-DE-FRANCE	0.53 [0.23 ; 0.74]	0.55 [0.26 ; 0.75]	0.81 [0.64 ; 0.90]
ILE-DE-FRANCE	0.45 [0.11 ; 0.69]	0.71 [0.48 ; 0.84]	0.69 [0.44 ; 0.83]
NORMANDIE	0.70 [0.46 ; 0.84]	0.83 [0.67 ; 0.91]	0.76 [0.55 ; 0.87]
NOUVELLE-AQUITAINE	0.55 [0.25 ; 0.75]	0.73 [0.52 ; 0.86]	0.89 [0.78 ; 0.94]
OCCITANIE	0.78 [0.59 ; 0.89]	0.61 [0.34 ; 0.79]	0.84 [0.69 ; 0.92]
PAYS-DE-LA-LOIRE	0.53 [0.23 ; 0.74]	0.57 [0.28 ; 0.76]	0.55 [0.26 ; 0.76]
PROVENCE-ALPES-COTE-D-AZUR	0.56 [0.26 ; 0.76]	0.62 [0.36 ; 0.80]	0.79 [0.61 ; 0.89]
